# Genomic prediction based on preselected single‐nucleotide polymorphisms from genome‐wide association study and imputed whole‐genome sequence data annotation for growth traits in Duroc pigs

**DOI:** 10.1111/eva.13651

**Published:** 2024-02-15

**Authors:** Yuling Zhang, Zhanwei Zhuang, Yiyi Liu, Jinyan Huang, Menghao Luan, Xiang Zhao, Linsong Dong, Jian Ye, Ming Yang, Enqin Zheng, Gengyuan Cai, Zhenfang Wu, Jie Yang

**Affiliations:** ^1^ College of Animal Science and National Engineering Research Center for Breeding Swine Industry South China Agricultural University Guangzhou China; ^2^ Guangdong Provincial Key Laboratory of Agro‐animal Genomics and Molecular Breeding South China Agricultural University Guangzhou China; ^3^ Guangdong Zhongxin Breeding Technology Co., Ltd Guangzhou China; ^4^ College of Animal Science and Technology Zhongkai University of Agriculture and Engineering Guangzhou China

**Keywords:** annotation, genomic prediction, growth traits, pigs

## Abstract

The use of whole‐genome sequence (WGS) data is expected to improve genomic prediction (GP) power of complex traits because it may contain mutations that in strong linkage disequilibrium pattern with causal mutations. However, a few previous studies have shown no or small improvement in prediction accuracy using WGS data. Incorporating prior biological information into GP seems to be an attractive strategy that might improve prediction accuracy. In this study, a total of 6334 pigs were genotyped using 50K chips and subsequently imputed to the WGS level. This cohort includes two prior discovery populations that comprise 294 Landrace pigs and 186 Duroc pigs, as well as two validation populations that consist of 3770 American Duroc pigs and 2084 Canadian Duroc pigs. Then we used annotation information and genome‐wide association study (GWAS) from the WGS data to make GP for six growth traits in two Duroc pig populations. Based on variant annotation, we partitioned different genomic classes, such as intron, intergenic, and untranslated regions, for imputed WGS data. Based on GWAS results of WGS data, we obtained trait‐associated single‐nucleotide polymorphisms (SNPs). We then applied the genomic feature best linear unbiased prediction (GFBLUP) and genomic best linear unbiased prediction (GBLUP) models to estimate the genomic estimated breeding values for growth traits with these different variant panels, including six genomic classes and trait‐associated SNPs. Compared with 50K chip data, GBLUP with imputed WGS data had no increase in prediction accuracy. Using only annotations resulted in no increase in prediction accuracy compared to GBLUP with 50K, but adding annotation information into the GFBLUP model with imputed WGS data could improve the prediction accuracy with increases of 0.00%–2.82%. In conclusion, a GFBLUP model that incorporated prior biological information might increase the advantage of using imputed WGS data for GP.

## 1 INTRODUCTION

Genomic prediction (GP) was initially proposed in 2001 (Meuwissen et al., 2001) and is a powerful tool for estimating genomic estimated breeding values (GEBV) using genome‐wide markers. Over the past decade, genomic selection (GS), primarily relied on single‐nucleotide polymorphism (SNP) chip data, has been successfully and widely used in plant (Hayes et al., 2013; Heffner et al., 2009) and livestock breeding (Erbe et al., 2012; Sonesson & Meuwissen, 2009). In addition, the significant reduction of sequencing costs allowed the incorporation of whole‐genome sequencing (WGS) in genomic selection (Daetwyler et al., 2014). Compared to SNP arrays, WGS data contain a significant number of genomic variants, including all or the majority of the causative mutations or SNPs that are in strong linkage disequilibrium (LD) with causative mutations affecting the traits, which is expected to be beneficial, and this was confirmed in two simulation studies (Iheshiulor et al., 2016; Meuwissen & Goddard, 2010). Some studies, nevertheless, have shown that using WGS data did not improve or increased slightly the prediction accuracy (van Binsbergen et al., 2015; Ye et al., 2019; Zhang et al., 2018). A possible reason is that only sequence variants very close to causative mutations could improve the accuracy of GP (van den Berg et al., 2016). According to Perez‐Enciso et al. (2015), rare SNPs and linkage disequilibrium also affect the prediction accuracy.

An alternate approach to employ all WGS data is to incorporate only causative mutations or SNPs in strong LD with causative mutations (van den Berg et al., 2016). One of the strategies is to supplement the available marker arrays by preselecting variants associated with traits of interest from WGS data. For instance, Brondum et al. (2015) proposed that the prediction accuracy could be improved by adding significant quantitative trait loci (QTL) or variants that were selected based on genome‐wide association studies (GWAS) using WGS data. Additionally, VanRaden et al. (2017) reported that adding preselected SNPs with the largest estimated effects could improve prediction accuracy. These preselected variants are known as prior biological information. In theory, any additional information that can affect the phenotype can be used as biological prior information for GP. Gene annotation (Gao et al., 2017), gene expression (Li et al., 2019), gene ontology (Edwards et al., 2016), proteome, and metabolome can all be used as prior biological information for GP. Considering the availability of genome annotation information, some studies have improved prediction accuracy by incorporating annotation information into prediction models. For instance, Nani et al. (2019) concluded that incorporating functional information into the predictive models could enhance the prediction of dairy bull fertility. Here we used the genomic feature best linear unbiased prediction (GFBLUP) model proposed by Edwards et al. (2016). GFBLUP is an extension of genomic best linear unbiased prediction (GBLUP). The GBLUP model using a single random effect. Whereas the GFBLUP model separates the total genomic components into two random genetic components (Ye et al., 2020).

In this study, two Duroc pig populations with different genetic backgrounds were used as validation population and other two Landrace and Duroc populations as prior discovery population to investigate the predictive performance of six growth traits under different scenarios. We used annotation information of the pig genome to divide imputed WGS data into different genomic classes, six of which were selected, including intergenic regions (IGR), intron regions (ITR), 3′ untranslated regions (3′UTR), synonymous (SYN), downstream (DOWN) and upstream (UP). We also preselected trait‐associated SNPs based on the GWAS results. Then, these different variant panels were added to the standard 50K and WGS data separately. The objectives of this study were (1) to assess the prediction of growth traits using 50K and WGS data, (2) to evaluate the predictive power of each genomic annotation class, and (3) to evaluate the predictive performance by incorporating different variant panels into GP models.

## 2 MATERIALS AND METHODS

### 2.1 Ethics approval

All experimental procedures involving animals in this study met the guidelines of the care and use of experimental animals established by the Chinese Ministry of Agriculture. Ethics approval for this study was given by the Ethics Committee of South China Agricultural University (SCAU, Guangzhou, China). Experimental animals were not anesthetized or euthanized.

### 2.2 Population and phenotypes

#### 2.2.1 Validation populations

The validation populations consisted of 5854 Duroc pigs, sampled from two breeding farms in Guangdong Wen's Foodstuffs Co., Ltd. (Guangdong, China). 3770 of American origin (AD) were born between 2013 and 2017, and 2084 of Canadian origin (CD) were born between 2015 and 2017. All animals were raised under the same feeding conditions. Phenotypic records included days to 100 kg (AGE), average daily gain (ADG), backfat thickness (BF), loin muscle area (LMA), loin muscle depth (LMD), and lean meat percentage (LMP). ADG and AGE were measured from 30 to 115 kg and then adjusted to 100 kg. AGE was adjusted to 100 kg using formula below (Tang et al., 2019):

$$\mathrm{AGE}\;\text{adjusted to}\;100\;\mathrm{kg}=\text{Measured}\;\mathrm{age}-\left(\frac{\text{Measured weight}-100\;\mathrm{kg}}{\text{Correction factor}}\right)$$

where correction factors (CF) are different for sire and dam, and the formulas are shown below:

$$\text{Sire}:\text{Correction factor}=\frac{\text{Measured weight}}{\text{Measured}\;\mathrm{age}}\times 1.826$$

$$\mathrm{Dam}:\text{Correction factor}=\frac{\text{Measured weight}}{\text{Measured}\;\mathrm{age}}\times 1.715$$

ADG adjusted to 100 kg was calculated by following equation (Tang et al., 2019):

$$\mathrm{ADG}\;\text{adjusted to}\;100\;\mathrm{kg}=\frac{100\;\mathrm{kg}}{\mathrm{AGE}\;\text{adjusted to}\;100\;\mathrm{kg}}$$

Phenotypes of BF, LMD and LMA were collected by experienced investigators from the 10th to 11th‐ribs of pigs at the weight of 100 ± 5 kg by an Aloka 500 V SSD B ultrasound (Corometrics Medical Systems, USA) (Suzuki et al., 2005), which used a diagnostic ultrasound system and transducers to obtain high‐resolution images and computer software to determine the LMA. BF and LMD adjusted to 100 kg were calculated as reported by the Canadian Center for Swine Improvement (http://www.ccsi.ca/Reports/Reports_2007/Update_of_weight_adjustment_factors_for_fat_and_lean_depth.pdf):

$$\mathrm{BF}\;\text{adjusted to}\;100\;\mathrm{kg}=\text{Measured}\;\mathrm{BF}\times \frac{A}{A+B\times \left(\text{Measured Weight}-100\right)}$$

where *A* and *B* are different for sire and dam, as follows:

Sire:A=13.47;B=0.1115

Dam:A=15.65;B=0.1566

LMD adjusted to 100 kg was calculated by following equation (Zhao et al., 2019):

$$\mathrm{LMD}\;\text{adjusted to}\;100\;\mathrm{kg}=\text{Measured}\;\mathrm{LMD}\times \frac{a}{a+b\times \left(\text{Measured Weight}-100\right)}$$

where *a* and *b* are different for sire and dam, as follows:

Sire:a=50.52;B=0.228

Dam:a=52.01;B=0.228

LMP was adjusted to 100 kg using formula below (Zhao et al., 2019):

LMPadjusted to100kg=61.21920−0.77665×BF+0.15239×LMD

Phenotypes were pre‐corrected for fixed effects using PREDICTF90 (Misztal et al., 2014). Corrected phenotypes were used as response variables in GP analyses.

#### 2.2.2 Prior discovery populations

In this study, prior discovery populations consisting of two pure breeds, including 294 Landrace and 186 Duroc, designated LL_GK and DD_GK. All pigs were reared on one farm at Guangdong Guangken Group Co., Ltd. (Guangdong, China) under similar feeding conditions. A subset of the most informative variants was preselected based on the GWAS results obtained from the prior discovery population. Phenotypic records included ADG, AGE, BF, LMA, and LMP.

### 2.3 Genotyping and imputation

Genotyping was conducted as described by Ding et al. (2018). The genomic DNA was extracted from ear tissue using standard protocols, and DNA quality was determined using electrophoresis and the ratios of light absorption (*A*_260/280_ and *A*_260/230_). The validation populations genotyping was performed using the GeneSeek Porcine 50K Chip (Neogen, Lincoln, NE, USA). The PLINK software (v1.90) (Purcell et al., 2007) was used for quality control (QC) at the following criteria: individual call rate >90%, SNP call rate >90%, minor allele frequencies (MAF) >5%, and *p* > 10^−6^ for the Hardy–Weinberg equilibrium test. Only SNPs located on the autosome chromosomes were retained in this study. After quality control, 35,563 SNPs for 3770 American Duroc pigs and 32,854 SNPs for 2084 Canadian Duroc pigs were retained. The prior discovery populations were genotyped using the CC1PorcineSNP50 BeadChip plus (Beijing Compass Agritechnology Co., Ltd., Beijing, China). After genotyping, imputation process of 50K genotypes in validation population and prior discovery population to WGS data was performed using SWIM. The SWIM is a pig haplotype reference panel, which was developed based on 2259 whole genome‐sequenced animals representing 44 pig breeds, and exhibited stable power in genotype imputation for 50K chip with an average concordance rate in excess of 96% and *r* ^2^ of 0.85 (Ding et al., 2023). After genotype imputation, the following quality control criteria were used to remove variants from the imputed WGS data: SNP call rate <90%, MAF <5%, and *p* < 10^−6^ for the Hardy–Weinberg equilibrium test. Moreover, SNPs located on sex chromosomes were excluded. In the validation population, 10,163,506 SNPs, 1,667,182 INDELs for 3770 American Duroc pigs, and 9,193,695 SNPs, 1,538,647 INDELs for 2084 Canadian Duroc pigs remained after QC. In the prior discovery population, 12,293,513 SNPs for Landrace pigs and 11,340,494 SNPs for Duroc pigs remained after QC.

### 2.4 Variant annotation

All filtered SNPs and INDELs from imputed whole‐genome variants in validation population and prior discovery population were annotated using an SNP annotation tool, SnpEff (Cingolani et al., 2012), which accepts variants in Variant Call Format. For annotation, the database containing genomic annotations for Sscrofa 11.1 (Ensembl release 99) was used. Based on their genomic location, whole SNPs and INDELs were partitioned into 18 and 19 different categories, respectively. Then, some genomic classes, such as splice variants, start and stop sites, were not considered due to their extremely low proportion. Finally, six major classes of genomic regions were considered: (1) 3′ untranslated regions (3′UTR), (2) downstream (DOWN), (3) upstream (UP), (4) intergenic regions (IGR), (5) intron regions (ITR), and (6) synonymous (SYN). Downstream and upstream refer to regions located 5‐kb downstream and 5‐kb downstream of genes. The 3′UTR variants are those located in the 3′ untranslated region. Intergenic variants refer to variants that are in an intergenic region. The intron variants are those located in the intron region. Synonymous variant means a sequence variant where there is no resulting change to the encoded amino acid. The number and proportion of variants annotated in various genomic regions are displayed in Table 1.

**TABLE 1:** Number of variants annotated in different genomic classes in validation population and prior discovery population.^a^

Variant type	Functional class	AD		CD		LL_GK		DD_GK	
		Number of variants	Proportion (%)	Number of variants	Proportion (%)	Number of variants	Proportion (%)	Number of variants	Proportion (%)
SNP	Intron variant	5,063,699	49.822	4,536,532	49.344	6,171,760	50.203	5,641,221	49.744
	Intergenic region	4,874,464	47.960	4,454,129	48.448	5,846,639	47.559	5,448,426	48.044
	Downstream gene variant	694,940	6.838	629,992	6.852	848,754	6.904	777,392	6.855
	Upstream gene variant	680,754	6.698	614,113	6.680	830,331	6.754	760,303	6.704
	Non coding transcript exon variant	122,663	1.207	108,803	1.183	149,484	1.216	135,046	1.191
	3′UTR variant	118,458	1.166	107,769	1.172	147,739	1.202	133,242	1.175
	Synonymous variant	45,768	0.450	41,937	0.456	55,699	0.453	51,551	0.455
	5′UTR variant	37,689	0.371	34,689	0.377	46,797	0.381	42,297	0.373
	Missense variant	16,481	0.162	14,588	0.159	20,820	0.169	18,875	0.166
	Splice region variant	9960	0.098	8945	0.097	12,335	0.100	11,192	0.099
	5 prime UTR premature start codon gain variant	3446	0.034	6075	0.066	8239	0.067	7443	0.066
	Splice donor variant	359	0.004	337	0.004	431	0.004	414	0.004
	All stop variants	330	0.003	295	0.003	400	0.003	370	0.003
	Splice acceptor variant	272	0.003	260	0.003	339	0.003	308	0.003
	Start lost variant	82	0.001	64	0.001	107	0.001	95	0.001
	Start retained variant	39	0.000	43	0.000	41	0.000	44	0.000
	Intragenic variant	0	0.000	145	0.002	720	0.006	266	0.002
	Initiator codon variant	0	0.000	7	0.000	7	0.000	8	0.000
INDEL	Intron variant	827,795	49.652	754,031	48.974				
	Intergenic region	809,033	48.527	756,694	49.147				
	Downstream gene variant	113,825	6.827	104,898	6.813				
	Upstream gene variant	111,063	6.662	102,256	6.642				
	3′UTR variant	19,836	1.190	18,442	1.198				
	Non coding transcript exon variant	19,558	1.173	17,762	1.154				
	5′UTR variant	5813	0.349	5455	0.354				
	Inframe variant	350	0.021	344	0.022				
	Frameshift variant	338	0.020	2784	0.181				
	Splice region variant	289	0.017	2310	0.150				
	Intragenic variant	70	0.004	31	0.002				
	Splice donor variant	31	0.002	626	0.041				
	Non coding transcript variant	30	0.002	33	0.002				
	Splice acceptor variant	11	0.001	607	0.039				
	Gene fusion	5	0.000	5	0.000				
	All stop variants	5	0.000	67	0.004				
	Start lost variant	2	0.000	19	0.001				
	Start retained variant	2	0.000	7	0.000				
	Exon loss variant	1	0.000	1	0.000				

^a^ Annotation of variants were performed based on the genome Sscrofa11.1 using SnpEff v5.1. Since some variants are located in several transcripts and thus be assigned to different regions, the cumulative value is more than 100%.

### 2.5 Genome‐wide association study

To measure LD levels in discovery and validation populations, we calculated the correlation coefficient (*r* ^2^) of alleles via PopLDdecay software (Zhang et al., 2019). Then, genetic distances between prior discovery population and validation population were calculated using an identity‐by‐state (IBS) similarity kinship matrix by PLINK. A genome‐wide association study (GWAS) was applied in the prior discovery population for growth traits using GCTA software (Yang, Lee, et al., 2011). The univariate mixed linear model is as follows:

$$y_{c}=1\mu +x_{i}\alpha_{i}+\mathrm{Ζg}+e,$$

where $y_{c}$ is a vector of the corrected phenotypes, μ is the intercept, 1 is a vector of ones, $x_{i}$ is a vector of genotypes, $\alpha_{i}$ is the effect of the ith sequence variant; Ζ is an incidence matrix for animals, g is the vector of random additive genetic effects of animals, following a normal distribution of $g-N\left(0,G\sigma_{g}^{2}\right)$, where *G* is the genomic relationship matrix (GRM) constructed from imputed WGS data and $\sigma_{g}^{2}$ is the variance explained by SNPs; $e-N\left(0,I\sigma_{e}^{2}\right)$, is the vector of random residual effects. Given that Bonferroni correction is a stringent criterion, false discovery rate (FDR) was used to determine the threshold *p* values of GWAS (Wang et al., 2017). FDR was set as 0.05, and the threshold *p* value was defined as *p* = FDR × *N*/*M*, where *N* is the number of SNPs with *p*‐value <0.05 in the results of GWAS and *M* is the total number of SNPs. Then, the significant SNPs were selected into the models to predict breeding value.

### 2.6 Preselection of SNPs based on the GWAS and annotation

We obtained six genomic class SNPs by annotating WGS data from prior discovery population and validation population. We also performed GWAS using WGS data from prior discovery population to obtain traits‐associated SNPs. Then, we incorporated these seven different variant panels (i.e., six annotations of variants and significant SNPs) into 50K chip data and WGS data of validation population. The GBLUP and GFBLUP models were used for GP to estimate the GEBV of growth traits with these different variant panels. When performing GFBLUP, common SNPs that were present on variant panels of the prior discovery population and WGS data of validation population were selected as final subsets of WGS data.

### 2.7 Statistical models

Two models were used to predict genomic EBV: GBLUP based on the GRM and GFBLUP that includes an additional genomic component for a set of variants associated with genomic features. For GBLUP, the datasets include 50K SNP chip data, imputed WGS data, and different genomic classes annotated from imputed WGS data. For GFBLUP, we used two strategies to evaluate the performance of the model. One strategy is to add different variant panels to 50K chip data. Another strategy is to add different variant panels to WGS data.

#### 2.7.1 Genomic BLUP

The GBLUP model was used to predict the genomic EBV based on a linear mixed model including only one random genomic effect:

$$y_{c}=1\mu +\mathrm{Ζg}+e,$$

where $y_{c}$ is a vector of the corrected phenotypes, μ is the overall mean, 1 is a vector of ones, g is the vector of additive genetic values, following a normal distribution of $g-N\left(0,G\sigma_{g}^{2}\right)$, where $\sigma_{g}^{2}$ is the additive genetic variance and G is the marker‐based GRM (VanRaden, 2008), which was performed using GCTA software (Yang, Lee, et al., 2011). Z is an incidence matrix linking g to $y_{c}$, and e is the vector of random residual effect, following a normal distribution of $e-N\left(0,I\sigma_{e}^{2}\right)$, where $\sigma_{e}^{2}$ is the residual variance. The prediction of breeding value with GBLUP model was performed using the R package EMMREML (Akdemir & Okeke, 2015).

#### 2.7.2 Genomic feature BLUP

The GFBLUP model was an extended BLUP including two random genetic effects:

$$y_{c}=1\mu +\mathrm{Ζf}+\mathrm{Zr}+e,$$

where $y_{c},1,\mu$, and e are the same as in GBLUP, f is the vector of genomic values captured by genetic markers associated with a genomic feature of interest, following a normal distribution of $f-N\left(0,G_{f}\sigma_{f}^{2}\right)$, r is the vector of genomic effects captured by the remaining set of genetic markers, following a normal distribution of $r-N\left(0,G_{r}\sigma_{r}^{2}\right)$, and Z is an incidence matrix linking f and r to $y_{c}$. The $G_{f}$ and $G_{r}$ were constructed similarly using only the genetic marker set defined by the genomic feature and the remaining set of markers, respectively. Since the computational resources of using two G matrices are too high, the two G matrices were combined into one G matrix to predict the genomic EBV:

$$G_{\text{Total}}=\lambda G_{f}+\left(1-\lambda \right)G_{r}$$

where $\lambda =\frac{\sigma_{f}^{2}}{\sigma_{f}^{2}+\sigma_{r}^{2}}$. $\sigma_{f}^{2}$ refers to the additive genetic variance captured by features, and $\sigma_{r}^{2}$ refers to the additive genetic variance captured by remained markers in the dataset. Variance components were estimated using the REML algorithm via GCTA software (Yang, Lee, et al., 2011).

**FIGURE 1:**
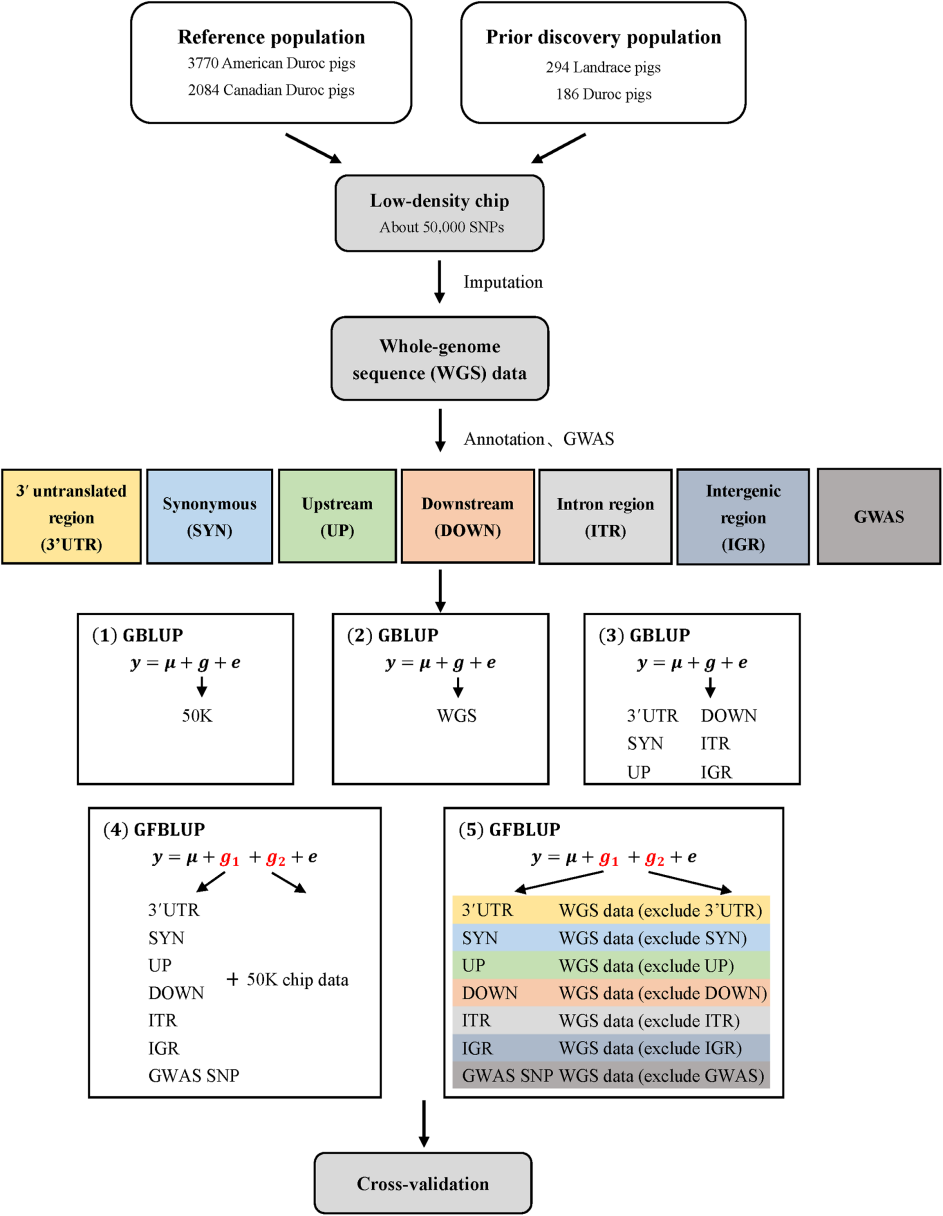
Flowchart of genomic prediction based on preselected SNPs from GWAS and imputed whole‐genome sequence data annotation.

In total, after SNPs and INDELs from imputed WGS data were annotated, five approaches were used to estimate breeding values for six growth traits. (1) GBLUP_50K which used the 50K SNP chip data to calculate the GRM (G). (2) GBLUP_WGS which used the imputed WGS data to calculate the GRM (G). (3) GBLUP which used different genomic classes generated from the WGS data to calculate the GRM (G) separately. (4) GFBLUP which used a SNP panel generated from the WGS data to construct G_1_ and the 50K SNP chip data to construct G_2_. (5) GFBLUP which used a SNP panel generated from the WGS data to construct G_1_ and the remaining of WGS to construct G_2_ (see Figure 1). It is worth noting that when method (4) was used for GP, the duplicated SNPs in two genetic components will be removed in SNP panels.

### 2.8 Evaluation of the accuracy of genomic prediction

The accuracy of the GP was assessed using a fivefold cross‐validation with one repetition. Briefly, the genotyped individuals were randomly divided into five groups of nearly equal size. One group was treated as the validation set, and the remaining four groups were used as the reference set. The cross‐validation procedure was then done five times to ensure that each group was validated once. The prediction accuracy was calculated as the correlation between the GEBV and their corrected phenotypes in the validation population. Finally, the average prediction accuracy values for the 25 cross‐validation round per trait were reported.

## 3 RESULTS

### 3.1 Statistical phenotypes and heritability

We collected phenotypic data from 5854 Duroc pigs from two different genetic backgrounds, including ADG, AGE, BF, LMA, LMD, and LMP. Descriptive statistic of the phenotypes and heritability are shown in Table S1. The coefficients of variation (CV) of six growth traits in American and Canadian Duroc pig ranged from 2.51% to 12.33% and 2.89% to 19.07%, respectively. The results show that a large variation of BF phenotypes in Canadian Duroc pig. The heritability of six growth traits ranged from 0.25 (BF and LMP) to 0.39 (LMA) in AD and from 0.21 (AGE and ADG) to 0.32 (LMD) in CD.

### 3.2 Variants annotation summary

We annotated imputed WGS data from the validation and prior discovery populations using SnpEff based on their physical positions. SNPs and INDELs were annotated into 18 and 19 different genomic classes (Table 1). The results showed that the most common annotation classes were intron regions (48%–50%) and intergenic variants (47%–49%), with approximately 6% each in upstream and downstream, approximately 1.5% in UTR, and 0–0.5% in other classes.

### 3.3 Genome‐wide association study in prior discovery population

**FIGURE 2:**
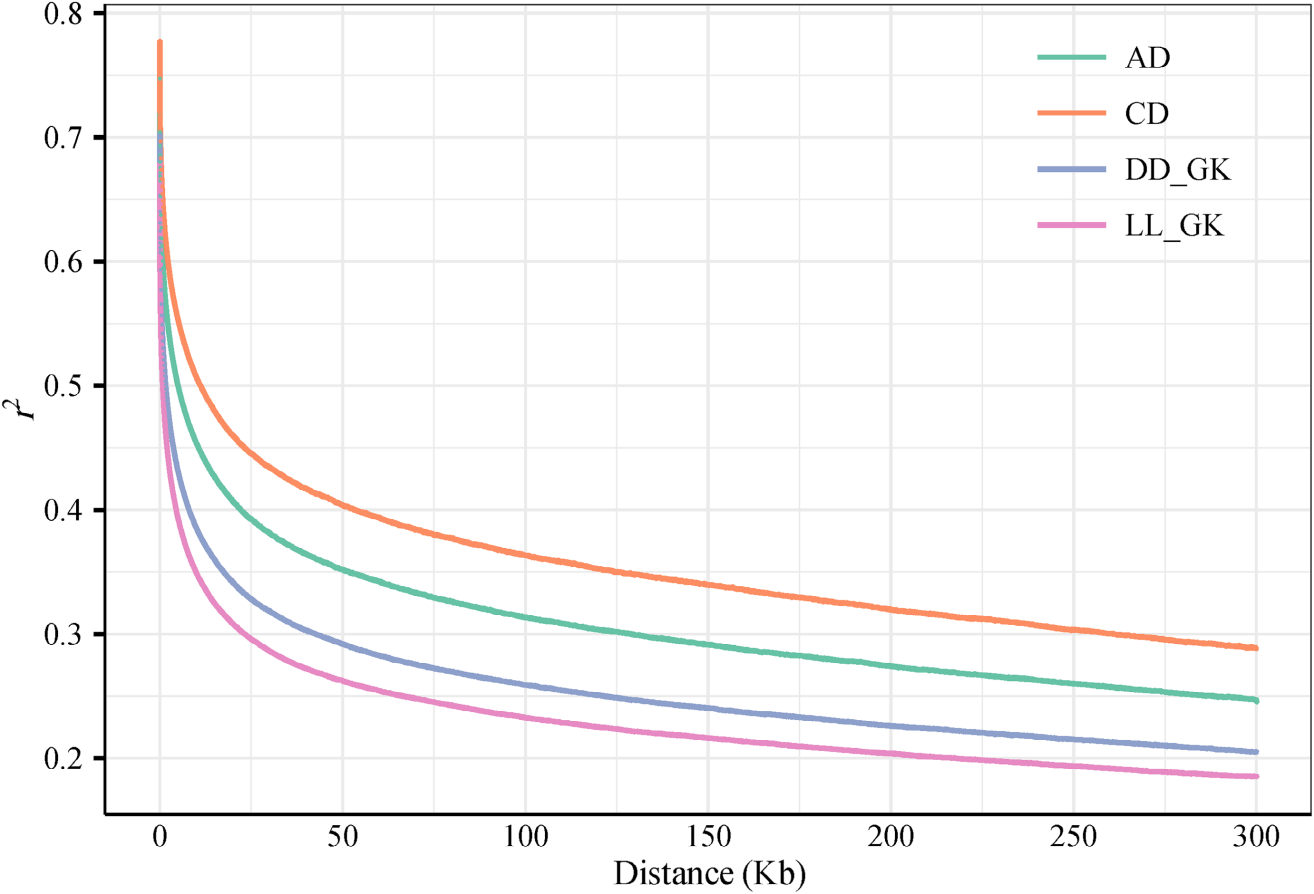
LD decay across the whole genome of prior discovery population and validation population. AD, American Duroc pig; CD, Canadian Duroc pig; LL_GK, Landrace pig from Guangdong Guangken Group Co., Ltd.; DD_GK, Duroc pig from Guangdong Guangken Group Co., Ltd.

We calculated the linkage disequilibrium in AD, CD, LL_GK, and DD_GK. We also calculated the IBS similarity matrix between prior discovery population and validation population. LD decay plot are shown in Figure 2. Different population have similar linkage disequilibrium decay patterns. The genetic distance between LL_GK and AD (CD) ranged from 0.6 to 1. The genetic distance between DD_GK and AD (CD) ranged from 0.65 to 1 (Figure S1). We then used the imputed‐WGS of prior discovery population to perform GWAS and set FDR to 0.05. The Manhattan plots are shown in Figures S2 and S3. Common SNPs that presented between GWAS results and WGS data of validation population were used preselected for subsequent GP.

### 3.4 Genomic prediction

#### 3.4.1 Comparison of the GBLUP method based on different classes

We examined the prediction accuracy of genomic classes and GBLUP_50K using the GBLUP model to determine which genomic classes were beneficial for GP of growth traits. The accuracy of GP with different genomic classes for the growth traits is presented in Table 2. We found that the prediction accuracy of different genome classes varied with traits, and there is only a small difference in the prediction accuracy among different genomic classes. For the six growth traits in two Duroc pig populations, the difference between the genomic class with the highest prediction accuracy and the lowest of INDEL ranged from 0.007 to 0.027. For all traits, using WGS data did not improve the prediction accuracy compared to GBLUP_50K. As compared to GBLUP_50K, genomic class SNPs also did not improve the prediction accuracy.

**TABLE 2:** Accuracy and standard error of genomic prediction for growth traits in two Duroc populations using different classes.

Validation population^a^	Class^b^	Variant type	ADG	AGE	BF	LMA	LMD	LMP
AD	GBLUP_50K	SNP	0.420 (0.005)	0.408 (0.005)	0.359 (0.006)	0.490 (0.005)	0.463 (0.005)	0.358 (0.007)
	GBLUP_WGS	SNP	0.408 (0.005)	0.396 (0.005)	0.357 (0.006)	0.486 (0.005)	0.458 (0.005)	0.355 (0.007)
		INDEL	0.407 (0.005)	0.395 (0.005)	0.355 (0.006)	0.487 (0.005)	0.455 (0.005)	0.352 (0.007)
	3′UTR	SNP	0.397 (0.005)	0.385 (0.005)	0.349 (0.006)	0.478 (0.005)	0.457 (0.005)	0.351 (0.006)
		INDEL	0.394 (0.005)	0.382 (0.005)	0.346 (0.006)	0.478 (0.005)	0.448 (0.005)	0.348 (0.007)
	DOWN	SNP	0.405 (0.005)	0.393 (0.005)	0.359 (0.005)	0.482 (0.005)	0.455 (0.004)	0.359 (0.006)
		INDEL	0.405 (0.005)	0.394 (0.005)	0.350 (0.006)	0.478 (0.005)	0.449 (0.005)	0.346 (0.006)
	IGR	SNP	0.408 (0.005)	0.396 (0.005)	0.348 (0.006)	0.477 (0.006)	0.448 (0.005)	0.341 (0.007)
		INDEL	0.399 (0.005)	0.388 (0.005)	0.349 (0.006)	0.474 (0.005)	0.443 (0.005)	0.339 (0.007)
	ITR	SNP	0.404 (0.005)	0.392 (0.005)	0.361 (0.006)	0.481 (0.005)	0.455 (0.005)	0.361 (0.007)
		INDEL	0.398 (0.005)	0.386 (0.005)	0.351 (0.006)	0.483 (0.006)	0.452 (0.005)	0.354 (0.007)
	SYN	SNP	0.399 (0.005)	0.387 (0.005)	0.352 (0.006)	0.478 (0.005)	0.456 (0.004)	0.356 (0.007)
	UP	SNP	0.406 (0.005)	0.394 (0.005)	0.359 (0.006)	0.485 (0.005)	0.455 (0.005)	0.360 (0.006)
		INDEL	0.404 (0.005)	0.392 (0.005)	0.355 (0.006)	0.477 (0.005)	0.448 (0.005)	0.356 (0.006)
CD	GBLUP_50K	SNP	0.315 (0.006)	0.310 (0.006)	0.376 (0.008)	0.390 (0.007)	0.394 (0.007)	0.392 (0.007)
	GBLUP_WGS	SNP	0.307 (0.007)	0.302 (0.007)	0.375 (0.008)	0.390 (0.007)	0.392 (0.007)	0.387 (0.007)
		INDEL	0.300 (0.007)	0.295 (0.007)	0.373 (0.007)	0.389 (0.007)	0.392 (0.007)	0.386 (0.007)
	3′UTR	SNP	0.304 (0.007)	0.302 (0.007)	0.364 (0.008)	0.397 (0.007)	0.391 (0.007)	0.384 (0.008)
		INDEL	0.296 (0.007)	0.292 (0.007)	0.373 (0.008)	0.401 (0.007)	0.399 (0.007)	0.394 (0.008)
	DOWN	SNP	0.312 (0.007)	0.308 (0.007)	0.369 (0.009)	0.394 (0.007)	0.392 (0.007)	0.384 (0.009)
		INDEL	0.301 (0.008)	0.298 (0.007)	0.370 (0.009)	0.398 (0.007)	0.395 (0.008)	0.388 (0.009)
	IGR	SNP	0.310 (0.007)	0.306 (0.006)	0.372 (0.007)	0.382 (0.007)	0.383 (0.007)	0.380 (0.007)
		INDEL	0.295 (0.007)	0.290 (0.007)	0.367 (0.007)	0.374 (0.008)	0.378 (0.007)	0.376 (0.007)
	ITR	SNP	0.300 (0.007)	0.295 (0.006)	0.367 (0.008)	0.396 (0.007)	0.390 (0.008)	0.383 (0.007)
		INDEL	0.291 (0.007)	0.285 (0.007)	0.366 (0.008)	0.391 (0.007)	0.388 (0.008)	0.384 (0.008)
	SYN	SNP	0.307 (0.007)	0.304 (0.007)	0.369 (0.009)	0.393 (0.006)	0.384 (0.007)	0.384 (0.009)
	UP	SNP	0.310 (0.007)	0.307 (0.007)	0.370 (0.009)	0.389 (0.007)	0.384 (0.008)	0.386 (0.008)
		INDEL	0.305 (0.007)	0.301 (0.007)	0.368 (0.009)	0.389 (0.007)	0.383 (0.007)	0.385 (0.008)

^a^ American Duroc pig (AD), Canadian Duroc pig (CD).

^b^ Genomic BLUP based on the 50K SNP panel (GBLUP_50K), Genomic BLUP based on the WGS data (GBLUP_WGS), Downstream (DOWN), Intergenic region (IGR), Intron region (ITR), Synonymous (SYN), Upstream (UP).

#### 3.4.2 GFBLUP method adding different variant panels to 50K chip data

Table 3 shows the accuracy of GP for the six growth traits adding different variant panels to 50K chip data in the two Duroc populations. Prediction accuracies obtained with GBLUP_50K in AD and CD for six growth traits ranged from 0.358 to 0.490 and 0.310 to 0.394, respectively. Compared with GBLUP_50K, adding different variant panels from imputed WGS variants to the 50K and treating them as two genetic components led to a slight reduction in prediction accuracy. The results showed no advantage of genome prediction using the GFBLUP method of adding annotation information or GWAS significant SNPs to the 50K.

**TABLE 3:** Accuracy and standard error of the genomic prediction adding different variant panel to 50K chip data.

Prior discovery population^a^	Validation population^b^	Class^c^	Variant type	ADG	AGE	BF	LMA	LMD	LMP
	AD	GBLUP_50K	SNP	0.420 (0.005)	0.408 (0.005)	0.359 (0.006)	0.490 (0.005)	0.463 (0.005)	0.358 (0.007)
LL_GK	AD	3′UTR	SNP	0.414 (0.005)	0.402 (0.005)	0.357 (0.006)	0.489 (0.005)	0.463 (0.005)	0.358 (0.007)
		DOWN	SNP	0.416 (0.005)	0.404 (0.005)	0.359 (0.006)	0.489 (0.005)	0.463 (0.005)	0.360 (0.007)
		IGR	SNP	0.417 (0.005)	0.404 (0.005)	0.357 (0.006)	0.488 (0.005)	0.460 (0.005)	0.353 (0.007)
		ITR	SNP	0.416 (0.005)	0.403 (0.005)	0.360 (0.006)	0.489 (0.005)	0.462 (0.005)	0.360 (0.007)
		SYN	SNP	0.414 (0.005)	0.402 (0.005)	0.358 (0.006)	0.490 (0.005)	0.464 (0.005)	0.360 (0.007)
		UP	SNP	0.417 (0.005)	0.405 (0.005)	0.359 (0.006)	0.491 (0.005)	0.464 (0.005)	0.361 (0.007)
		GWAS	SNP	0.408 (0.005)	0.393 (0.005)	0.356 (0.006)	0.479 (0.005)		0.354 (0.007)
DD_GK	AD	3′UTR	SNP	0.414 (0.005)	0.402 (0.005)	0.357 (0.006)	0.489 (0.005)	0.462 (0.005)	0.358 (0.007)
		DOWN	SNP	0.416 (0.005)	0.404 (0.005)	0.360 (0.006)	0.490 (0.005)	0.462 (0.005)	0.360 (0.007)
		IGR	SNP	0.416 (0.005)	0.404 (0.005)	0.357 (0.006)	0.488 (0.005)	0.458 (0.005)	0.353 (0.007)
		ITR	SNP	0.415 (0.005)	0.403 (0.005)	0.360 (0.006)	0.489 (0.005)	0.461 (0.005)	0.359 (0.007)
		SYN	SNP	0.414 (0.005)	0.402 (0.005)	0.358 (0.006)	0.489 (0.005)	0.463 (0.005)	0.360 (0.007)
		UP	SNP	0.416 (0.005)	0.404 (0.005)	0.360 (0.006)	0.491 (0.005)	0.463 (0.005)	0.361 (0.007)
		GWAS	SNP	0.404 (0.005)	0.387 (0.005)	0.355 (0.006)	0.479 (0.005)		0.351 (0.007)
AD	AD	3′UTR	SNP	0.414 (0.005)	0.402 (0.005)	0.358 (0.006)	0.490 (0.005)	0.464 (0.005)	0.359 (0.007)
			INDEL	0.415 (0.005)	0.403 (0.005)	0.358 (0.006)	0.492 (0.005)	0.463 (0.005)	0.358 (0.007)
		DOWN	SNP	0.416 (0.005)	0.404 (0.005)	0.361 (0.006)	0.491 (0.005)	0.464 (0.005)	0.361 (0.007)
			INDEL	0.418 (0.005)	0.406 (0.005)	0.360 (0.006)	0.492 (0.005)	0.464 (0.005)	0.360 (0.007)
		IGR	SNP	0.417 (0.005)	0.405 (0.005)	0.357 (0.006)	0.488 (0.005)	0.459 (0.005)	0.353 (0.007)
			INDEL	0.416 (0.005)	0.404 (0.005)	0.357 (0.006)	0.489 (0.005)	0.459 (0.005)	0.353 (0.007)
		ITR	SNP	0.415 (0.005)	0.403 (0.005)	0.361 (0.006)	0.490 (0.005)	0.462 (0.005)	0.360 (0.007)
			INDEL	0.415 (0.005)	0.402 (0.005)	0.359 (0.006)	0.490 (0.005)	0.461 (0.005)	0.359 (0.007)
		SYN	SNP	0.414 (0.005)	0.402 (0.005)	0.359 (0.006)	0.490 (0.005)	0.465 (0.005)	0.361 (0.007)
		UP	SNP	0.417 (0.005)	0.404 (0.005)	0.361 (0.006)	0.492 (0.005)	0.464 (0.005)	0.362 (0.007)
			INDEL	0.417 (0.005)	0.405 (0.005)	0.359 (0.006)	0.492 (0.005)	0.463 (0.005)	0.361 (0.007)
	CD	GBLUP_50K	SNP	0.315 (0.006)	0.310 (0.006)	0.376 (0.008)	0.390 (0.007)	0.394 (0.007)	0.392 (0.007)
LL_GK	CD	3′UTR	SNP	0.313 (0.007)	0.308 (0.006)	0.373 (0.008)	0.398 (0.007)	0.397 (0.007)	0.392 (0.008)
		DOWN	SNP	0.316 (0.007)	0.312 (0.006)	0.375 (0.009)	0.396 (0.007)	0.396 (0.007)	0.392 (0.008)
		IGR	SNP	0.315 (0.007)	0.309 (0.006)	0.376 (0.008)	0.387 (0.007)	0.391 (0.007)	0.390 (0.007)
		ITR	SNP	0.311 (0.006)	0.306 (0.006)	0.376 (0.008)	0.394 (0.007)	0.394 (0.007)	0.392 (0.008)
		SYN	SNP	0.314 (0.007)	0.310 (0.006)	0.375 (0.009)	0.397 (0.007)	0.395 (0.007)	0.392 (0.008)
		UP	SNP	0.317 (0.007)	0.312 (0.006)	0.375 (0.009)	0.394 (0.007)	0.394 (0.007)	0.393 (0.008)
		GWAS	SNP	0.309 (0.006)	0.298 (0.007)	0.367 (0.008)	0.375 (0.007)		0.383 (0.007)
DD_GK	CD	3′UTR	SNP	0.312 (0.007)	0.308 (0.006)	0.372 (0.008)	0.399 (0.007)	0.397 (0.007)	0.390 (0.008)
		DOWN	SNP	0.316 (0.007)	0.311 (0.006)	0.374 (0.008)	0.397 (0.007)	0.395 (0.007)	0.391 (0.008)
		IGR	SNP	0.313 (0.006)	0.307 (0.006)	0.374 (0.008)	0.387 (0.007)	0.390 (0.007)	0.388 (0.007)
		ITR	SNP	0.310 (0.006)	0.304 (0.006)	0.374 (0.008)	0.395 (0.007)	0.392 (0.007)	0.390 (0.007)
		SYN	SNP	0.315 (0.007)	0.311 (0.006)	0.374 (0.008)	0.397 (0.007)	0.394 (0.007)	0.391 (0.008)
		UP	SNP	0.316 (0.007)	0.311 (0.006)	0.374 (0.008)	0.395 (0.007)	0.394 (0.007)	0.391 (0.008)
		GWAS	SNP	0.303 (0.006)	0.290 (0.006)	0.363 (0.008)	0.379 (0.007)		0.373 (0.008)
CD	CD	3′UTR	SNP	0.313 (0.007)	0.309 (0.006)	0.374 (0.008)	0.399 (0.007)	0.399 (0.007)	0.392 (0.008)
			INDEL	0.311 (0.007)	0.306 (0.006)	0.379 (0.008)	0.401 (0.007)	0.402 (0.007)	0.397 (0.008)
		DOWN	SNP	0.316 (0.007)	0.312 (0.006)	0.375 (0.008)	0.397 (0.007)	0.396 (0.007)	0.391 (0.008)
			INDEL	0.312 (0.007)	0.308 (0.007)	0.375 (0.008)	0.397 (0.007)	0.398 (0.007)	0.391 (0.008)
		IGR	SNP	0.313 (0.007)	0.308 (0.006)	0.375 (0.007)	0.388 (0.007)	0.391 (0.007)	0.388 (0.007)
			INDEL	0.309 (0.007)	0.304 (0.006)	0.374 (0.007)	0.387 (0.007)	0.391 (0.007)	0.388 (0.007)
		ITR	SNP	0.310 (0.006)	0.305 (0.006)	0.375 (0.008)	0.395 (0.007)	0.394 (0.007)	0.391 (0.007)
			INDEL	0.308 (0.006)	0.302 (0.006)	0.374 (0.008)	0.395 (0.007)	0.394 (0.007)	0.390 (0.008)
		SYN	SNP	0.315 (0.007)	0.311 (0.006)	0.375 (0.008)	0.398 (0.007)	0.396 (0.007)	0.392 (0.008)
		UP	SNP	0.316 (0.007)	0.312 (0.006)	0.375 (0.008)	0.395 (0.007)	0.394 (0.008)	0.392 (0.008)
			INDEL	0.313 (0.007)	0.308 (0.006)	0.374 (0.008)	0.394 (0.007)	0.394 (0.008)	0.392 (0.008)

^a^ LL_GK, Landrace pig from Guangdong Guangken Group Co., Ltd.; DD_GK, Duroc pig from Guangdong Guangken Group Co., Ltd.

^b^ American Duroc pig (AD), Canadian Duroc pig (CD).

^c^ Genomic BLUP based on the 50K SNP panel (GBLUP_50K), Downstream (DOWN), Intergenic region (IGR), Intron region (ITR), Synonymous (SYN), Upstream (UP).

#### 3.4.3 GFBLUP method adding different variant panels to WGS data

We also investigated the prediction accuracy of GFBLUP that considered WGS data and different variant panels from WGS data as two random variance components. To prevent the reuse of the markers, we removed the duplicate SNP from WGS data. The results are presented in Table 4. Compared with GBLUP_WGS, the improvement of GP accuracy for AD and CD was of 0.00% to 1.41% and 0.00% to 2.82%, respectively. For most scenarios, GFBLUP using annotation information outperformed GBLUP_WGS for six growth traits in both Duroc pig populations, while some scenarios reduced the prediction accuracy, e.g., for BF trait in the AD population, the prediction accuracy of 3′UTR SNP using LL_GK and GBLUP_WGS was 0.356 and 0.357, respectively. Compared with GBLUP_WGS, GFBLUP using GWAS based on WGS data did not improve prediction accuracy. We found that for ADG and AGE trait, the prediction accuracy of GFBLUP model using LL_GK annotation information was better than using AD annotation information when AD was used as the validation population. Previous study pointed out that using the same data for discovery and training resulted in biased prediction (Veerkamp et al., 2016). When the genomic relationships between the reference and validation sets are low, the benefit of using prioritized SNPs for GP is larger (MacLeod et al., 2016). According to de Las Heras‐Saldana et al. (2020), the selection of predictive SNPs was based on their association with the phenotype in relatively unrelated individuals, their contribution to prediction accuracy should be less affected by relatedness.

**TABLE 4:** Accuracy and standard error of the genomic prediction adding different variant panel to WGS data.

Prior discovery population^a^	Validation population^b^	Class^c^	Variant type	ADG	AGE	BF	LMA	LMD	LMP
	AD	GBLUP_WGS	SNP	0.408 (0.005)	0.396 (0.005)	0.357 (0.006)	0.486 (0.005)	0.458 (0.005)	0.355 (0.007)
			INDEL	0.407 (0.005)	0.395 (0.005)	0.355 (0.006)	0.487 (0.005)	0.455 (0.005)	0.352 (0.007)
LL_GK	AD	3′UTR	SNP	0.408 (0.005)	0.396 (0.005)	0.356 (0.006)	0.487 (0.005)	0.460 (0.005)	0.356 (0.007)
		DOWN	SNP	0.410 (0.005)	0.398 (0.005)	0.359 (0.006)	0.487 (0.005)	0.459 (0.005)	0.358 (0.007)
		IGR	SNP	0.411 (0.005)	0.399 (0.005)	0.357 (0.006)	0.486 (0.005)	0.457 (0.005)	0.353 (0.007)
		ITR	SNP	0.411 (0.005)	0.399 (0.005)	0.359 (0.006)	0.487 (0.005)	0.459 (0.005)	0.357 (0.007)
		SYN	SNP	0.409 (0.005)	0.397 (0.005)	0.357 (0.006)	0.489 (0.005)	0.462 (0.005)	0.358 (0.007)
		UP	SNP	0.411 (0.005)	0.399 (0.005)	0.359 (0.006)	0.489 (0.005)	0.461 (0.005)	0.359 (0.007)
		GWAS	SNP	0.401 (0.005)	0.388 (0.005)	0.357 (0.006)	0.475 (0.005)		0.350 (0.007)
DD_GK	AD	3′UTR	SNP	0.408 (0.005)	0.396 (0.005)	0.357 (0.006)	0.487 (0.005)	0.460 (0.005)	0.356 (0.007)
		DOWN	SNP	0.409 (0.005)	0.398 (0.005)	0.359 (0.006)	0.488 (0.005)	0.459 (0.005)	0.357 (0.007)
		IGR	SNP	0.410 (0.005)	0.399 (0.005)	0.358 (0.006)	0.486 (0.005)	0.457 (0.005)	0.354 (0.007)
		ITR	SNP	0.410 (0.005)	0.398 (0.005)	0.359 (0.006)	0.487 (0.005)	0.458 (0.005)	0.356 (0.007)
		SYN	SNP	0.408 (0.005)	0.396 (0.005)	0.357 (0.006)	0.488 (0.005)	0.460 (0.005)	0.357 (0.007)
		UP	SNP	0.410 (0.005)	0.398 (0.005)	0.359 (0.006)	0.489 (0.005)	0.460 (0.005)	0.359 (0.007)
		GWAS	SNP	0.396 (0.005)	0.378 (0.005)	0.353 (0.006)	0.474 (0.006)		0.349 (0.007)
AD	AD	3′UTR	SNP	0.408 (0.005)	0.396 (0.005)	0.357 (0.006)	0.488 (0.005)	0.461 (0.005)	0.357 (0.007)
			INDEL	0.407 (0.005)	0.395 (0.005)	0.354 (0.006)	0.489 (0.005)	0.457 (0.005)	0.354 (0.007)
		DOWN	SNP	0.410 (0.005)	0.398 (0.005)	0.360 (0.006)	0.488 (0.005)	0.460 (0.005)	0.358 (0.007)
			INDEL	0.411 (0.005)	0.399 (0.005)	0.357 (0.006)	0.489 (0.005)	0.458 (0.005)	0.355 (0.007)
		IGR	SNP	0.410 (0.005)	0.398 (0.005)	0.358 (0.006)	0.487 (0.005)	0.458 (0.005)	0.355 (0.007)
			INDEL	0.408 (0.005)	0.396 (0.005)	0.355 (0.006)	0.487 (0.006)	0.455 (0.005)	0.352 (0.007)
		ITR	SNP	0.410 (0.005)	0.398 (0.005)	0.358 (0.006)	0.487 (0.005)	0.458 (0.005)	0.355 (0.007)
			INDEL	0.408 (0.005)	0.396 (0.005)	0.355 (0.006)	0.487 (0.006)	0.455 (0.005)	0.352 (0.007)
		SYN	SNP	0.408 (0.005)	0.396 (0.005)	0.358 (0.006)	0.489 (0.005)	0.462 (0.005)	0.358 (0.007)
		UP	SNP	0.410 (0.005)	0.398 (0.005)	0.360 (0.006)	0.489 (0.005)	0.461 (0.005)	0.360 (0.007)
			INDEL	0.410 (0.005)	0.398 (0.005)	0.356 (0.006)	0.489 (0.005)	0.457 (0.005)	0.356 (0.007)
	CD	GBLUP_WGS	SNP	0.307 (0.007)	0.302 (0.007)	0.375 (0.008)	0.390 (0.007)	0.392 (0.007)	0.387 (0.007)
			INDEL	0.300 (0.007)	0.295 (0.007)	0.373 (0.007)	0.389 (0.007)	0.392 (0.007)	0.386 (0.007)
LL_GK	CD	3′UTR	SNP	0.309 (0.007)	0.305 (0.006)	0.371 (0.008)	0.401 (0.007)	0.397 (0.007)	0.389 (0.008)
		DOWN	SNP	0.312 (0.007)	0.308 (0.006)	0.373 (0.008)	0.397 (0.007)	0.395 (0.007)	0.388 (0.008)
		IGR	SNP	0.309 (0.007)	0.304 (0.006)	0.374 (0.007)	0.390 (0.007)	0.391 (0.007)	0.387 (0.007)
		ITR	SNP	0.307 (0.006)	0.302 (0.006)	0.374 (0.008)	0.394 (0.007)	0.392 (0.007)	0.388 (0.007)
		SYN	SNP	0.310 (0.007)	0.306 (0.006)	0.374 (0.008)	0.399 (0.007)	0.394 (0.007)	0.389 (0.008)
		UP	SNP	0.312 (0.007)	0.308 (0.006)	0.374 (0.008)	0.396 (0.007)	0.393 (0.007)	0.389 (0.008)
		GWAS	SNP	0.304 (0.007)	0.293 (0.007)	0.367 (0.007)	0.376 (0.007)		0.379 (0.007)
DD_GK	CD	3′UTR	SNP	0.308 (0.007)	0.304 (0.006)	0.371 (0.008)	0.400 (0.007)	0.396 (0.007)	0.387 (0.008)
		DOWN	SNP	0.311 (0.007)	0.307 (0.007)	0.373 (0.008)	0.397 (0.007)	0.393 (0.007)	0.387 (0.008)
		IGR	SNP	0.307 (0.007)	0.302 (0.006)	0.374 (0.007)	0.391 (0.007)	0.391 (0.007)	0.386 (0.007)
		ITR	SNP	0.307 (0.007)	0.302 (0.006)	0.373 (0.007)	0.393 (0.007)	0.391 (0.007)	0.387 (0.007)
		SYN	SNP	0.311 (0.007)	0.308 (0.007)	0.373 (0.008)	0.398 (0.007)	0.393 (0.007)	0.388 (0.008)
		UP	SNP	0.311 (0.007)	0.307 (0.006)	0.373 (0.008)	0.395 (0.007)	0.392 (0.007)	0.388 (0.008)
		GWAS	SNP	0.298 (0.006)	0.282 (0.007)	0.360 (0.008)	0.381 (0.007)		0.368 (0.008)
CD	CD	3′UTR	SNP	0.309 (0.007)	0.305 (0.007)	0.373 (0.008)	0.400 (0.007)	0.397 (0.007)	0.389 (0.008)
			INDEL	0.302 (0.007)	0.298 (0.007)	0.376 (0.008)	0.399 (0.007)	0.399 (0.007)	0.393 (0.008)
		DOWN	SNP	0.312 (0.007)	0.308 (0.007)	0.373 (0.008)	0.397 (0.007)	0.394 (0.008)	0.388 (0.008)
			INDEL	0.304 (0.007)	0.300 (0.007)	0.372 (0.008)	0.396 (0.007)	0.395 (0.007)	0.387 (0.008)
		IGR	SNP	0.307 (0.007)	0.302 (0.006)	0.374 (0.007)	0.392 (0.007)	0.391 (0.007)	0.387 (0.007)
			INDEL	0.300 (0.007)	0.295 (0.007)	0.371 (0.007)	0.389 (0.007)	0.390 (0.007)	0.385 (0.007)
		ITR	SNP	0.307 (0.007)	0.302 (0.006)	0.374 (0.007)	0.393 (0.007)	0.391 (0.007)	0.387 (0.007)
			INDEL	0.300 (0.007)	0.295 (0.007)	0.371 (0.007)	0.390 (0.007)	0.390 (0.007)	0.385 (0.007)
		SYN	SNP	0.311 (0.007)	0.307 (0.007)	0.374 (0.008)	0.399 (0.007)	0.395 (0.007)	0.388 (0.008)
		UP	SNP	0.312 (0.007)	0.308 (0.006)	0.374 (0.008)	0.395 (0.007)	0.393 (0.008)	0.388 (0.008)
			INDEL	0.305 (0.007)	0.300 (0.007)	0.371 (0.008)	0.392 (0.007)	0.391 (0.008)	0.387 (0.008)

^a^ LL_GK, Landrace pig from Guangdong Guangken Group Co., Ltd.; DD_GK, Duroc pig from Guangdong Guangken Group Co., Ltd.

^b^ American Duroc pig (AD), Canadian Duroc pig (CD).

^c^ Genomic BLUP based on the 50K SNP panel (GBLUP_50K), Genomic BLUP based on the WGS data (GBLUP_WGS), Downstream (DOWN), Intergenic region (IGR), Intron region (ITR), Synonymous (SYN), Upstream (UP).

## 4 DISCUSSION

Given advances in high‐throughput technology and the availability of annotation information, it is possible to incorporate annotation information and GWAS into GPs (Do et al., 2015; Morota et al., 2014; Nani et al., 2019; Xu et al., 2020). In this study, we applied two models, GBLUP and GFBLUP, to the genetic assessment of six growth traits in two Duroc pig populations using different methods. First, we compared the prediction performance of 50K chip data and WGS data. Second, we annotated six different genomic classes from imputed WGS data and evaluated their prediction performance. Finally, we investigated the predictive performance of different variant panels when adding them to 50K and WGS data, respectively.

### 4.1 Genomic prediction using 50K chip and WGS data

In this study, we investigated the prediction accuracy of the 50K chip and WGS data. In principle, the use of WGS data in GP leads to higher accuracy, because WGS data contain causal variants that affect the traits. Meuwissen and Goddard (2010) demonstrated in a simulation study that GP using WGS data is superior to high‐density markers. However, in this study, the prediction accuracy using WGS data was slightly reduced compared to 50K data (see Table 2). Similarly, other researchers on feed efficiency, reproduction and production traits in pigs (Song et al., 2019; Zhang et al., 2018), conformation trait in cattle (Frischknecht et al., 2018; Song et al., 2018), and carcass trait in chicken (Ye et al., 2019) showed no or only small improvements in prediction accuracy when using WGS data compared to chip data. There are several factors that may affect the prediction accuracy. First, the assumption of genomic selection is that QTL affecting quantitative traits are in linkage disequilibrium (LD) with at least one of the markers in the high‐density genome‐wide markers. According to the simulation study by Macleod et al. (2014), low LD has a significant advantage in using sequence data. In WGS data, a large number of SNPs are not associated with QTLs affecting the trait of interest or SNPs with high LD can lead to redundancy and noise and thus affect the prediction accuracy. Second, the WGS data were obtained by imputation, and consequently, imputation errors are generated, which affect the accuracy of GP. Van den Berg et al. (2017) reported that increasing the number of imputation errors reduces prediction accuracy. Overall, when using imputed WGS data for genome prediction, the accuracy of prediction depends on LD pruning, genotype imputation, and the genetic architecture of traits.

### 4.2 Genomic partitioning and prediction accuracy of different classes

In this study, we partitioned different genomic classes according to the functional annotation of the variants. After the partitioning, we investigated the predictive performance of different genomic classes of variants, expecting to select the genomic class with the best predictive performance for further GP. It is also an important question which parts of the genome contribute more to the genetic variation of complex traits. The annotation results showed that most of the variant was located in intron and intergenic regions (approximately 98%). According to the annotation results for cattle by Bhuiyan et al. (2018), 99% of the variants are located in intron and intergenic regions. However, there was only a small difference in prediction accuracy between the different genomic classes. For instance, in the SNP set of the AD population, the difference in prediction accuracy between IGR and ITR was only 0.004 for ADG trait. First, we controlled for an equal number of variants in different genomic classes in this study. In general, the number of markers plays an important role in the prediction accuracy of GP (Daetwyler et al., 2010). The contribution of different genomic classes is largely linear in their number of SNPs. Xu et al. (2020) indicated that UTR accounted for only 0.39%, with the lowest prediction accuracy. Second, the regulation of complex traits is diverse. It has been reported that about 90% of SNPs associated with traits in human are not in coding regions (Kavanagh et al., 2013). Hindorff et al. (2009) suggested that 45% of the trait‐associated SNPs from GWAS were intronic and 43% were intergenic. Yang, Manolio, et al. (2011) indicated that SNPs within or near genes account for more variation than SNPs between genes. According to MacLeod et al. (2016), using imputed sequence variants from coding and regulatory regions improved the accuracy of GP compared to HD (high density) SNP array. These studies suggest that different regions of the genomic contribute differently to the genetic variation of traits.

### 4.3 Comparison of GFBLUP and GBLUP models

In this study, we compared two different models, GBLUP and GFBLUP. We investigated the performance of GFBLUP model based on variants annotation and GWAS using two strategies, i.e., adding each variant panel obtained from annotation and GWAS to 50K and WGS data, respectively. Our results indicated that GFBLUP which adding annotation information to WGS yielded approximately 2% higher GP accuracy compared to standard GBLUP for growth traits (Table 4). In terms of model, both GBLUP and GFBLUP use all genomic variants, but GFBLUP has the advantage of allowing different weights to be assigned to variants in different genomic relationships depending on estimated genomic parameters, providing a better understanding of the genetic structure of traits (Edwards et al., 2016). Moreover, other researchers have used GFBLUP to improve prediction accuracy. Fang et al. (2017) demonstrated that the accuracy of GP was enhanced with GFBLUP compared to conventional GBLUP when using imputed sequence variants in Holstein and Jersey cattle. Similarly, Song et al. (2019) reported that GFBLUP achieved around 1% to 2% greater accuracy than GBLUP for reproduction and production traits based on WGS data in pigs. However, not all scenarios can improve prediction accuracy using GFBLUP, depending on the different strategies and the genetic architecture of traits. Our results showed that GFBLUP adding annotation information to 50K showed no improvement or a slight decrease in GP accuracy (Table 3). The pig 50K chip was designed based on common variants and can explain most of the genetic variance of the phenotype, but some rare variants cannot be excluded to explain significant variance. SNPs that are not related to analyzed traits were added to 50K chip data may introduce noise and result in biased to GP. On the other hand, the annotation in this study was based on the site of the variants without considering their LD. SNPs that are in LD between different classes may affect prediction accuracy. In addition, the accuracy of GP is also affected by population. We compared the prediction accuracy of using independent prior discovery population unrelated to the validation population and using validation population to select prior information. Our results showed that for AGE trait, prediction accuracy yielded approximately 0.2% higher accuracy of GP in AD population when using an independent prior discovery population unrelated to the validation population. Moghaddar et al. (2019) showed that when SNPs are selected from the same data set as those used for prediction, GP of breeding values is likely to be biased. Bias could be due to selectively use of random event information, but it may also result from not properly considering population structure or otherwise using selective data. Veerkamp et al. (2016) used preselected variants from GWAS with WGS data in Holstein‐Friesian cattle and found that the stringent selection of variants resulted in more biased GP. They suggested that the bias may come from the overlap between discovery and validation data due to the relationship between discovery and validation animals. MacLeod et al. (2016) also demonstrated that using prioritized SNPs for GP is more beneficial when the relationships between the reference and validation sets are lower. According to de Las Heras‐Saldana et al. (2020), since the SNPs are selected based on association with the phenotype of relatively unrelated individuals, their contribution to prediction accuracy should be less influenced by relatedness.

Some studies have demonstrated that using prior biological knowledge to performed GP can improve the prediction accuracy (Edwards et al., 2016; MacLeod et al., 2016). In this study, we found that adding preselected GWAS SNPs from the WGS data did not improve prediction accuracy, which agrees with the results of Veerkamp et al. (2016). Compared to using preselected SNPs from GWAS, using preselected SNPs from specific genomic regions resulted in higher prediction accuracy for all traits. In addition to GWAS and annotation information, GFBLUP models can easily incorporate prior information from different sources, such as biological pathways and other omics data. Using different prior information for GP results in different prediction accuracies. Ye et al. (2020) showed that using genomic features preselected from multi‐omics data is a feasible strategy to improve the power of GP.

## 5 CONCLUSIONS

This study investigated the prediction accuracy of different genomic classes obtained by annotation and incorporated the annotation information into the GFBLUP model for GP of six growth traits in two Duroc pig populations. We noted that a small difference between genomic regions with respect to predictive ability. Different genomic regions obtained by genome annotation had little effect on the prediction accuracy of the traits investigated here, which may differ for other traits, depending on the genetic architecture of the trait. While adding annotation information to the WGS data can improve the prediction accuracy. GFBLUP model incorporating prior biological information such as annotation information might increase the advantage of using imputed WGS data for GP.

## CONFLICT OF INTEREST STATEMENT

The authors declare that there is no conflict of interest.

## Supporting information


Figure S1

Figure S2

Figure S3

Table S1

## Data Availability

The SNP genotyping data containing variant information in validation population and prior discovery population are not publicly available because the genotyped animals belong to commercial breeding companies, but they can be obtained from the corresponding author under reasonable requirements.
