# Supplementary figures and images for: Genomic prediction based on preselected single‐nucleotide polymorphisms from genome‐wide association study and imputed whole‐genome sequence data annotation for growth traits in Duroc pigs
Source: Evol Appl. 2024 Feb 15;17(2):e13651. doi: 10.1111/eva.13651 (PMC10868536; doi:10.1111/eva.13651)

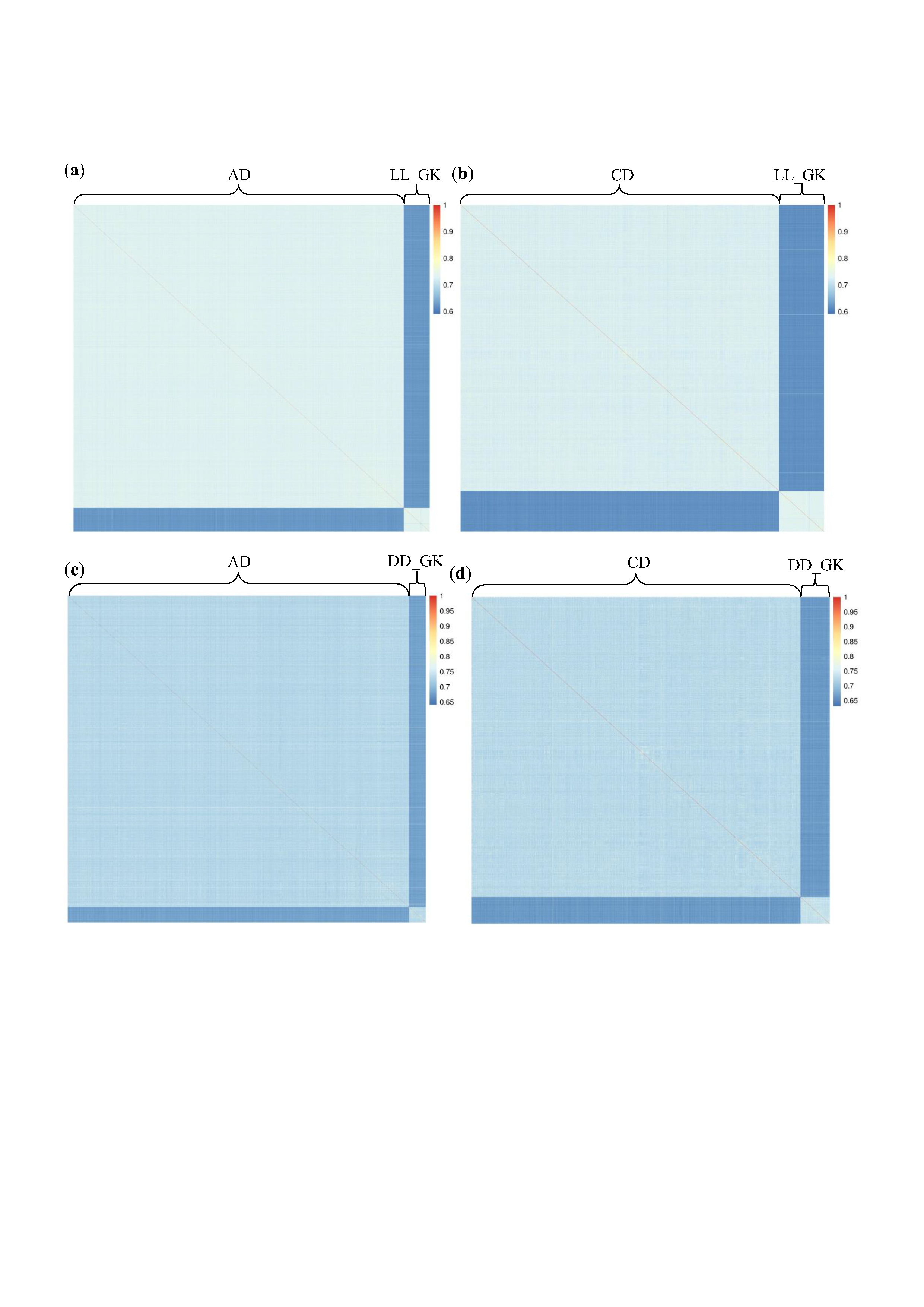

Supplement: Supplementary file 1 — Figure S1 [file EVA-17-e13651-s004.tif]

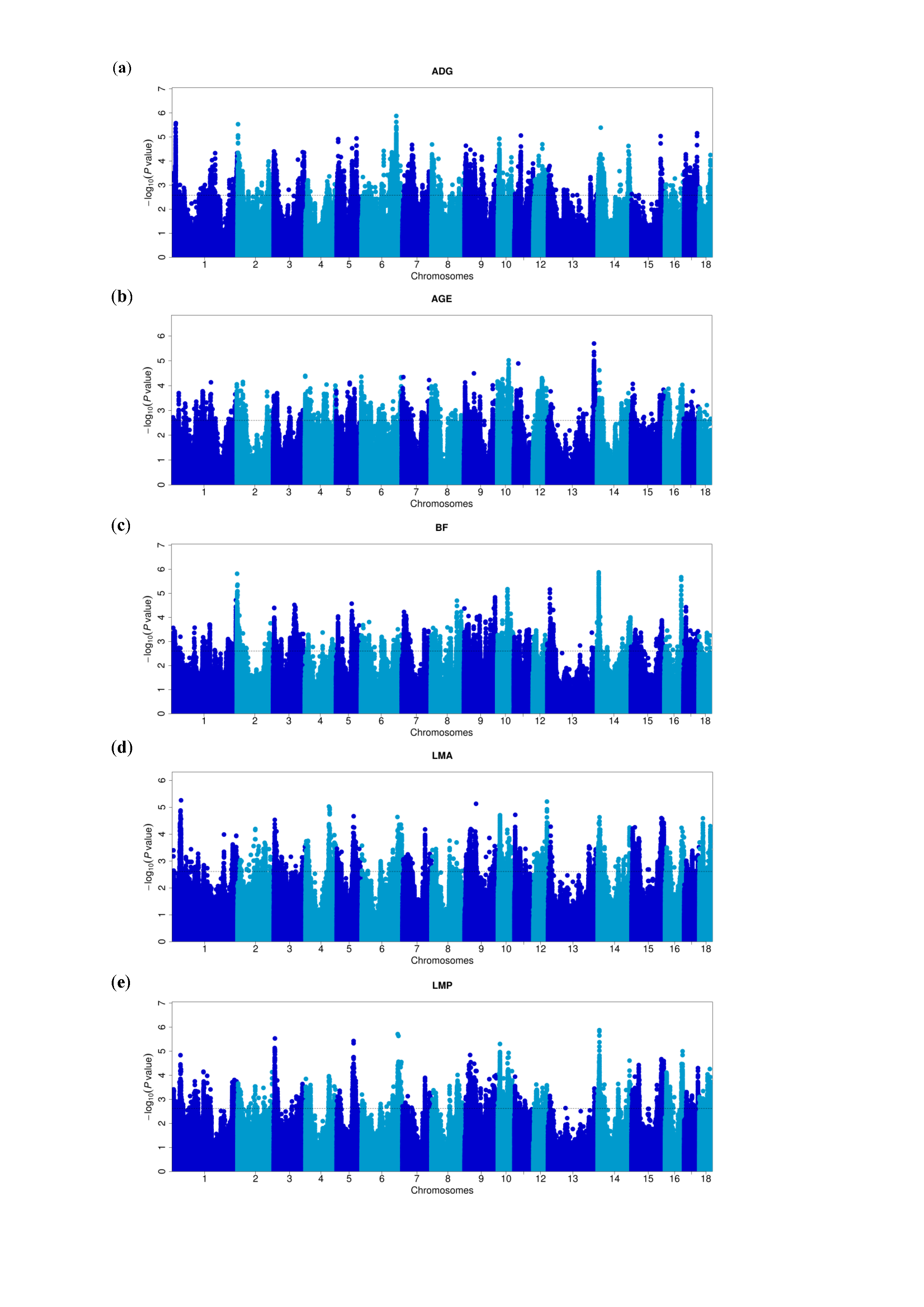

Supplement: Supplementary file 2 — Figure S2 [file EVA-17-e13651-s001.tif]

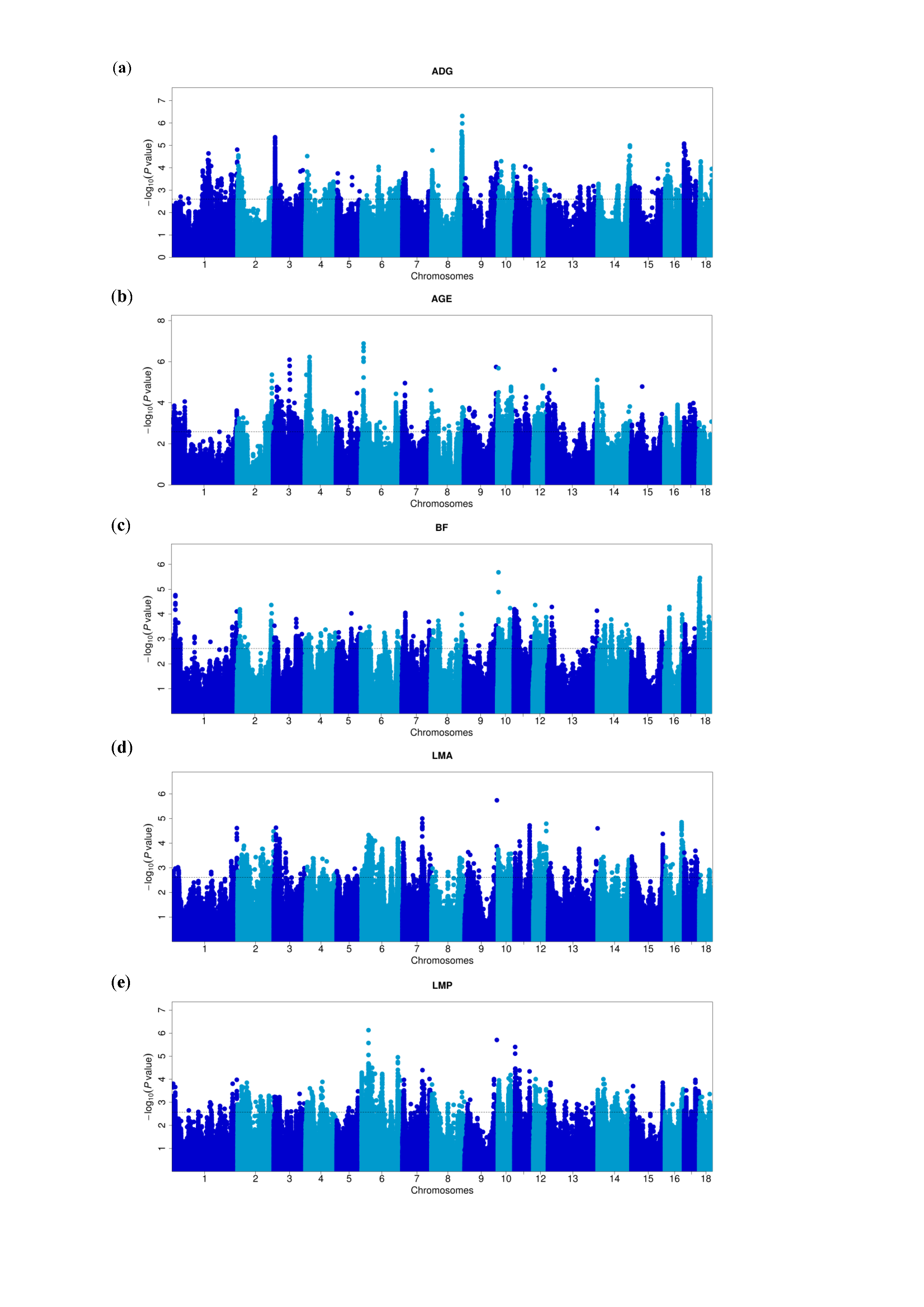

Supplement: Supplementary file 3 — Figure S3 [file EVA-17-e13651-s002.tif]
