# Supplementary material for: Genomic prediction based on preselected single‐nucleotide polymorphisms from genome‐wide association study and imputed whole‐genome sequence data annotation for growth traits in Duroc pigs
Source: Evol Appl. 2024 Feb 15;17(2):e13651. doi: 10.1111/eva.13651 (PMC10868536; doi:10.1111/eva.13651)
Supplement: Supplementary file 4 — Table S1 [file EVA-17-e13651-s003.docx]

**Table S1** **Number of phenotypic records and heritability for six growth traits in American Duroc pigs and Canadian Duroc pigs**

| Population^1^ | Trait^2^ | N | Mean ± SD | Max | Min | CV (%) | h^2^ (±SE) |
| --- | --- | --- | --- | --- | --- | --- | --- |
| AD | AGE (day) | 3770 | 159.09±8.44 | 193.32 | 128.8 | 5.30 | 0.31±0.02 |
|  | ADG (g) | 3770 | 619.43±32.7 | 758.54 | 509 | 5.28 | 0.30±0.02 |
|  | BF (mm) | 3770 | 8.97±1.11 | 17.91 | 6.09 | 12.33 | 0.25±0.02 |
|  | LMA (cm^2^) | 3770 | 38.77±3.51 | 51.56 | 23.69 | 9.05 | 0.39±0.02 |
|  | LMD (mm) | 3770 | 52.40±3.72 | 65 | 37.3 | 7.09 | 0.35±0.02 |
|  | LMP (%) | 3770 | 55.81±1.40 | 60.53 | 50.08 | 2.51 | 0.25±0.02 |
| CD | AGE (day) | 2084 | 160.88±11.48 | 205.96 | 127.82 | 7.13 | 0.21±0.03 |
|  | ADG (g) | 2084 | 613.60±43.22 | 769.05 | 478.73 | 7.04 | 0.21±0.03 |
|  | BF (mm) | 2084 | 9.65±1.84 | 17.31 | 5.1 | 19.07 | 0.26±0.03 |
|  | LMA (cm^2^) | 2084 | 35.81±3.63 | 48.84 | 25.4 | 10.13 | 0.31±0.03 |
|  | LMD (mm) | 2084 | 47.85±3.76 | 61.2 | 5.1 | 7.85 | 0.32±0.03 |
|  | LMP (%) | 2084 | 54.41±1.57 | 59.81 | 49.47 | 2.89 | 0.27±0.03 |

N, number of observations; SD, standard deviation; Max, maximum value; Min, minimum value; CV, coefficient of variation; h^2^, heritability; SE, standard error.

^1^ American Duroc pig (AD), Canadian Duroc pig (CD).

^2^ Days to 100kg (AGE), Average daily gain (ADG), Backfat thickness (BF), Loin muscle area (LMA), Loin muscle depth (LMD) and Lean meat percentage (LMP).
